# Functional Dependency in Mexico: Measurement Issues and Policy Challenges

**DOI:** 10.34172/ijhpm.2020.248

**Published:** 2020-12-19

**Authors:** Pablo Villalobos Dintrans, Emmanuel González Bautista

**Affiliations:** ^1^Programa Centro Salud Pública, Facultad de Ciencias Médicas, Universidad de Santiago, Santiago, Chile.; ^2^National Institute of Geriatrics, Mexico City, Mexico.

**Keywords:** Functional Capacity, Disability, Long-term Care, Measurement, Metrics, Mexico

## Abstract

**Background:** Different definitions have been used to measure functional dependency (FD) in Mexico. This study aims to explore if different definitions of FD lead to low consistency between the estimations of its prevalence. Accurate estimations of FD are useful to estimate the potential demand for long-term care (LTC) services in the country.

**Methods:** A literature review including documents with estimations on the number or prevalence of dependents in Mexico with national representativeness between 2000 and 2019 was performed as well as estimations of different definitions of FD, using the National Study on Health and Aging in Mexico (ENASEM).

**Results:** There is a lack of consensus on the definition of FD. Among the most frequently used terms to define FD are "disability" and "dependency." The heterogeneity of definitions results in a wide range of estimations of the demand for LTC. Methodological choices can lead to important differences in FD prevalence estimations. Results from ENASEM 2001 show that FD prevalence could range from 13% to 35% in people 60+; sex prevalences also vary when using different ways to measure FD.

**Conclusion:** Besides the highlighted issues in calculating FD in the population, Mexico should consider broadening the assessment of FD, including people with dementia and younger populations. Although the literature search is not systematic, it helps exemplifying the current issues when measuring FD in Mexico. A consensual definition of dependency is required to have a more accurate estimated demand for LTC. Having good data sources is not enough when dissimilar estimations of an indicator like dependency result from the same study. Wide heterogeneity in estimations of dependency could be an obstacle to inform public policies during the construction of a care system in Mexico.

## Background

**Key Messages**

** Implications for policy makers**

- Results highlight the importance of having information on functional dependency (FD) but also a consensus definition on how to measure it.
- Policy-makers should understand the policy implications of using different measurements definitions for FD.
- Long-term care (LTC) policies should consider people in need of services outside the group of older people.
- Despite the methodological issues arising from different definitions, the increasing demand for LTC services calls for a coordinated public policy response.

** Implications for the public**

Results shows that addressing the situation of long-term care (LTC) needs in Mexico requires an urgent and comprehensive response. The article highlights that evidence-based policy-making is required; considering the results would give a more accurate sense of the magnitude of the problem in the country. A consensus definition will not only help with this definition of the problem, but also increase transparency to the population. The country should move towards the implementation of a LTC system to provide services to this increasing demand.

Population aging in Mexico is happening at a fast pace. According to the latest United Nations estimates,^1^ the percentage of people over the age of 60, which was 5.4% in 1950, has doubled (11.2% in 2020) and will double again in the next 30 years (22.6% in 2050). This age group is projected to constitute almost 40% of the population in 2100, with the “oldest-old” (aged 85+) experiencing the fastest growth rates. Population aging, together with the burden of the life-course socio-economic disadvantage and chronic diseases are the perfect cocktail to boost the rates of disability and functional dependency (FD) in a given population cohort.^2-6^

 Understanding long-term care (LTC) needs in the Mexican population is crucial in the context of rapid aging with high rates of chronic diseases. LTC refers to activities carried out by others so that people who have had a significant and permanent loss of intrinsic capacity can maintain a level of functional capacity consistent with their basic rights, fundamental freedoms and dignity.^7^ The definition stresses the need to measure levels of FD as a potential estimation for the LTC demand.

 Mexico is among the few countries in Latin America and the Caribbean with data available to estimate the prevalence of FD at the national level.^8^ Statistics and information are fundamental during the design and implementation of evidence-based public policies.^9^ Despite this advantage (the country has several databases that allow estimating indicators of disability and FD), the lack of consensus for the definition and a standard methodology to measure FD may lead to inaccuracy when informing stakeholders and decision-makers. The question “How many dependents are there in Mexico?” is currently difficult to answer. The biggest problem does not seem to be the lack of data, but the absence of a standard definition, which may lead to multiple possible answers.

 Therefore, our study aims to answer how many dependents are there in Mexico and to explore how different definitions of FD lead to high or low consistency between estimations of its prevalence.

## Methods

###  Literature Review

 The analysis was carried out using documents that allowed developing a profile of FD in Mexico. We searched within online academic databases (PubMed and Google Scholar), and Google, to also span “grey literature,” such as reports from public and private institutions. For the search, key terms “Mexico,” “dependency,” “functionality” and “activities of daily life” were considered, including both documents in Spanish and English, from the year 2000 to information published in September 2019.

 The following inclusion criteria were considered: (*i*) document reporting statistics on the number or prevalence of dependents in Mexico; (*ii*) data with national representativeness; (*iii*) statistics on people living in the Mexican territory. Consequently, articles published before the year 2000,^10,11^ those reporting information based on samples without national representativeness,^12-14^ and studies on the population of Mexicans living abroad were excluded.^15,16^

 The first round of search yielded 27 documents. A new search stage was carried out to complete the list using the snowball sampling methodology, looking for new documents that could meet the criteria within the bibliographic references of each of the documents on the initial list.^17^ The final list included 32 documents and considers articles in academic journals, theses, and books.^6,8,18-46^

 Given that most of the selected document used the National Study on Health and Aging in Mexico (ENASEM) as their primary source of information (26 out of 32), the list was contrasted with the report publications reported by the ENASEM website (under the “Functionality” area), to ensure that no relevant information was missing.^47^ ENASEM is a longitudinal study with national representation for adults 50 years of age and older in Mexico, intended to analyze health and social determinants in the aged population. It started in 2001, and the last round of available data was collected in 2015.Once the documents were selected, each one was analyzed, and its information systematized, extracting reference, source of the data, the population of analysis, dependency-related concepts used, definition or instrument used to measure dependency, and results (prevalence or number of dependents).

###  ENASEM Estimations

 As a complement to the literature review, an empirical exercise is shown, using data from the ENASEM. Different ways to estimate FD are proposed, to exemplify that, despite being able to calculate a number, the methodological choices faced by researchers generate different results that are not whimsical when used to make policies. Seven FD definitions are measured, based on two dimensions: type of activity (basic activities of daily living, BADL vs instrumental activities of daily living, IADL), and type of limitation (difficulty vs help to perform activities).

- Definition 1: difficulty or inability to perform at least one BADL
- Definition 2: Help to perform (from spouse or other) at least one BADL
- Definition 3: difficulty or inability to perform at least one IADL
- Definition 4: Help to perform (from spouse or other) at least one IALD
- Definition 5: difficulty or inability to perform at least one BADL or one IADL
- Definition 6: help to perform (from spouse or other) at least one BADL or one IADL
- Definition 7: difficulty or inability OR help to perform (from spouse or other) at least one BADL or one IADL

The list of activities are extracted directly from the survey’s questionnaire and include:

- BADL: walking, bathing, eating, getting in and out of bed, using the toilet
- IADL: preparing a hot meal, shopping, taking medication, managing money.

## Results

 Based on the literature review, we find that there are three main factors driving the answers to the question “how many dependents are there in Mexico?”:

1. The approach used to measure FD: disability (BADLs, IADLs) or other disease-related concepts (frailty, dementia)
2. The definition of the age groups (60+, 65+, 70+, 75+)
3. The data source (ENASEM, National Survey on Health and Nutrition [ENSANUT], others)

The reviewed documents use different terms to define FD. The term “disability” is the one more often associated with dependency, along with other concepts related to functionality (dependency, capacity, impairment). We recognize the International Classification of Functioning (ICF) conceptual framework for disability, but none of the included documents refered to a ICF classification, so the definition of disability is limited to the “activity” domain of such framework.^48^

 Table shows that dependency prevalence reported by different studies range between 2% and 75% of the study population. The wide range of estimations for dependency is explained by the difference in data sources, concepts, and population of analysis. The table shows a lack of a consensus on how to measure FD. Researchers have to make several methodological decisions that contribute to the heterogeneity of the results reported around a single indicator (ie, FD prevalence): the set of activities to consider, the criteria for defining dependency, and finally, the way in which results are reported.

**Table:** Summary of Results From the Analysis of Selected Documents

**Data Sources**	ENASEM, ENSANUT, ENUT, Oportunidades Program database, Population and Housing Census
**Concepts Used to Define FD**	Disability/BADL disability /IADL disability Mobility/Mobility limitations Dependency/Funtional dependency/Care dependency Funcionality/Functionality limitation/Functional ability/Functional performance /Functional impairment/Functional problem Need for help (problem/difficulty to perform) BADL/IADL
**Other Related concepts Used**	Frailty Cognitive impairment/Cognitive functionality Dementia
**Population of Analysis**	50+; 60+; 65+; 70+; 80+
**Range of Results**	FD prevalence (all studies): 2%-74.8% Total number of people with FD (all studies): 2.8-8.5 millions FD prevalence by age groups: 50+: 10.7%-62%, 60+: 3%-26.9%, 65+: 3.3%-24%, 70+: 30.9%-44%, 80+: 37.5%-44%

Abbreviations: ENASEM, National Study on Health and Aging in Mexico; ENSANUT, National Survey on Health and Nutrition; ENUT, National Survey on the Use of Time; FD, functional dependency; IADL, instrumental activities of the daily living; BADL, basic activities of the daily living. Authors elaboration based on^6,8,18-46^.

**Figure 1:**
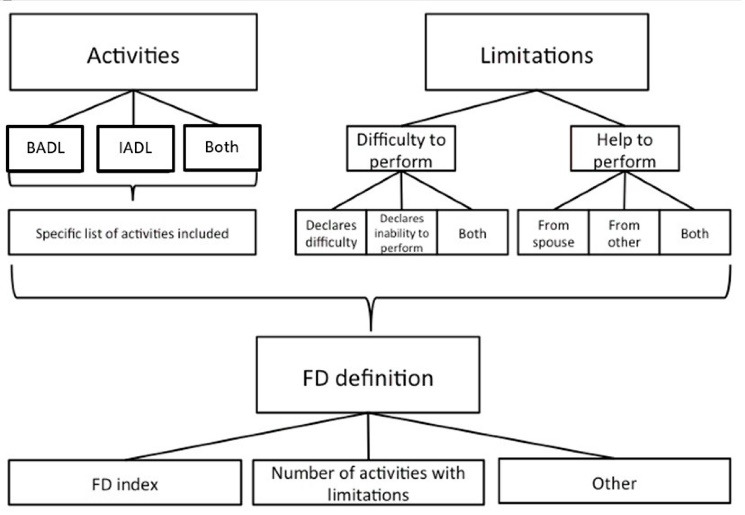
Decision Tree for Calculating FD Using the ENASEM. Abbreviations: ENASEM, National Study on Health and Aging in Mexico; FD, functional dependency; IADL, instrumental activities of the daily living; BADL, basic activities of the daily living.

 This wide range of results hold even when looking at studies using the same data source (see Supplementary file 1 for a summary of studies^3,13,19,20,21,28,34,36^ using the ENASEM). These methodological choices required to calculate FD are shown in Figure 1.

 Researchers must decide whether to calculate basic and instrumental limitations separately (versus, for example, building an index with both), and which questions to use in each case. As shown in Supplementary file 1, most have chosen to use the Katz index for BADLs and the Lawton and Brody index for IADL. Despite these coincidences, differences are observed in the type of activities used (eg, including difficulties in toileting and dressing as activities). When looking at the criterion to define dependency, most studies choose to construct a binary variable, based on the reported limitations to carry out some of the selected activities. In this case, the differences arise when trying to define what constitutes a limitation: reporting difficulty, requesting help, or answering the inability of carrying out the activity. Moreover, when reporting the results, studies usually use the prevalence of limitations in BADL and IADL. Additionally, studies present other indices,^20,22,35,42^ calculate the incidence,^39,44^ or report the number of dependents.^8^ Studies also differ in whether they include figures for the overall population and/ or separate the estimates by sex and age.

**Figure 2:**
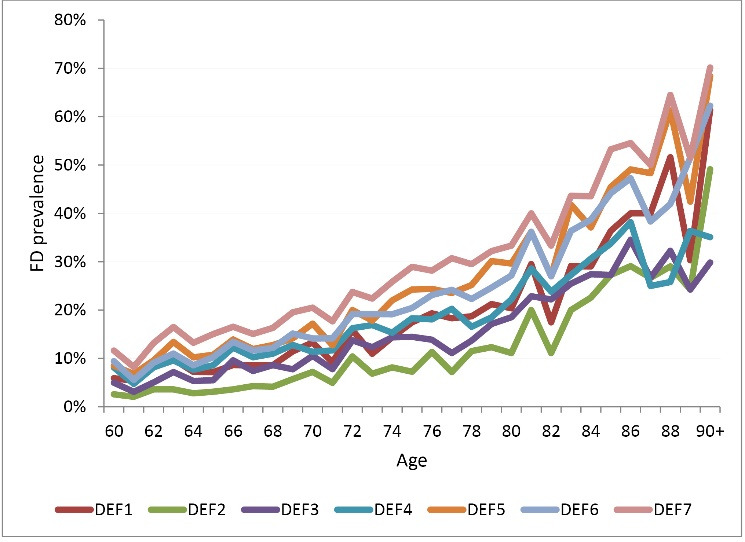
FD Age-Prevalences (60+) for Several Definitions Using the ENASEM 2001. Abbreviations: ENASEM, National Study on Health and Aging in Mexico; FD, functional dependency.

 Finally, the definitions previously proposed were calculated using the ENASEM 2001.^47^ These seven definitions present potential alternatives for measuring FD, although as can be checked in Supplementary file 1, other alternatives have been adopted by researchers using the ENASEM (including dressing, the use of continence, among others). Figure 2 shows that age prevalences for people 60+ differ considerably when using one definition or another.

 Prevalences range from 13.5% (definition 1) to 21.27% (definition 7). These numbers imply an estimation of the total number of people 60+ with FD ranging from 913 000 to almost 1.5 million people, based on the number of people 60+ in the 2000 census (6 948 457).^49^

**Figure 3:**
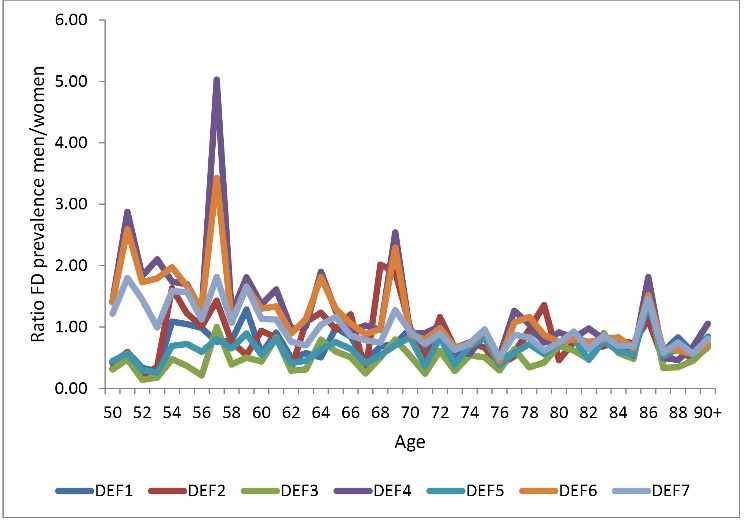
Sex Ration of FD Prevalences for Several Definitions Using the ENASEM 2001. Abbreviations: ENASEM, National Study on Health and Aging in Mexico; FD, functional dependency.

 However, the subestimation of the problem is not the only issue arising when using different definitions. Other dimensions of the analysis can be affected too. For example, Figure 3 shows the ratio of men/women FD prevalence using the same seven definitions.

 In this case, conclusions can drastically differ depending on the definition used. Results are particularly sensitive to the inclusion of difficulty to perform IADL. Policy recommendations using one or another definition can have huge impacts on certain groups, even though policy-makers decide to adopt an “evidence-based” approach.

## Discussion

 Results show the methodological choices researchers must face when dealing with measuring FD in a country with rich datasets available. They also highlight how the lack of a definition creates a problem when trying to use these results for policy-making.

 A first issue relates to the theoretical definition of FD. The problem of what FD means is evident when trying to measure the concept, but also notions more semantically distant — such as frailty, cognitive impairment, or dementia—were found in studies. Different theoretical frameworks for disability/dependency lead to different ways of measuring, and consequently, different estimates of FD. According to our results, the definition based on help to perform at least one BADL can be seen as a lower bound for the estimation of FD in the Mexican population because it is consistently the lower prevalence across the age range. This example shows the problem of lacking a definition of FD from a policy perspective.

 This finding raises another question, regarding the extent of the definition, in particular, whether people with dementia should be considered dependents in Mexico. The inclusion of mental health problems and dementia is an important topic to be considerer when establishing a national definition for FD, as well as when defining elegibility in a LTC system. Many LTC systems have moved into including dementia as an explicit component in their FD definitions,^50-52^ as well as countries in Latin America, such as Uruguay and Chile.^53-55^

 Second, a discussion on the target population of the analysis and the policy regarding FD is needed. On the one hand, although all studies focus on calculating dependency in the elderly population, several age thresholds were used. Interestingly, the reviewed documents generally assume that dependency and age are linked *vis-a-vis*. As already pointed out, there is a practical problem that researchers face when defining “old people” using chronological age as a criterion.^56-58^ For measuring dependency and especially from a decision-making perspective, the issue is relevant since the lack of definition for “older people” adds to the lack of definition for “dependency,” exacerbating the problems of comparability of studies and quantification of the problem. On the other hand, restricting the target population to older people ignores the fact that there are dependents who do not belong to this age group, and that they could represent a significant share of the dependents. For example, estimates for Chile show that about 40% of dependents are under 65 years of age^59^; in Uruguay, more than 60% of the LTC system beneficiaries are younger than 60 years.^60^ This topic is relevant, particularly considering that the country has surveys and information to include these populations into the analyses. For example, the ENSANUT, asks questions on difficulty to perform activities of daily living in children, adolescents, and adults.^61^

 Third, researchers should consider the data sources when presenting and interpreting their results. The Mexican case is important for other countries because it illustrates the relevance of having more and better information (solving the first-order problem of data scarcity), but also highlights the importance of moving towards a single definition and measurement tool (solving the second-order problem of a lack of standard measurement for FD). This step has been key for countries that have recently implemented and reformulated their LTC systems, such as Germany and South Korea.^50,51,62^ In Mexico, the availability of the ENASEM is crucial to allow research about dependency. However, it is not enough to answer pending public policy questions because of the lack of consistency obtained from studies using the same data source (eg, ENASEM). Also, the magnitude of FD in nursing homes and other institutions is a key figure currently lacking and it might cause underestimation of the people with FD when considering only community dwelling population. Creating a national registry for institutionalized people should be among the first actions towards a LTC system in Mexico.

 Finally, the study gives interesting insights but also has some limitations. Although we used a standardized search process, it does not constitute a systematic review. The methodological difficulty of carrying out systematic reviews in areas such as care dependency and FD comes from the diversity of terms and definitions.^63^ For instance, a medical subject heading (MeSH) term for care or FD has not been included yet. On the other hand, although the list of articles may not be exhaustive, the objective of the analysis — showing how the diversity of definitions, databases, and measurement instruments can be an obstacle to the generation of public policies — is achieved.

## Conclusion

 This study found that variations in the estimated magnitude of the dependents within the same country could be due to: (*i*) the definition or concept used; (*ii*) how this concept is operationalized (measurement tool); (*iii*) the age of the population of analysis and; (*iv*) the data source.

 The article poses a question that, at first glance, seems relatively simple: how many dependents are in Mexico? Based on the various studies reporting statistics of dependency in Mexico, we found multiple answers, and their wide heterogeneity represents a serious challenge to advance in the construction of policies in the area. For example, if we select the Mexican population aged 65+ today—roughly 13.8 million^64^—, the number of people who could potentially require LTC services varies between 2.8 and 8.5 million. This enormous range of results represents a huge problem to design policies and prioritize the discussion around LTC in the country.^65^ Using a lower-bound estimation to calculate the potential demand for LTC services could result in 5.5 million people failing to access services.

 Finally, our study reveals a series of challenges for Mexico in terms of measuring dependency and generating a LTC system. In this context, our results emphasize the importance of adopting a consensus standard definition of dependency for the country. This implies several challenges that need to be addressed. First, the need to advance in the elaboration of national definitions and instruments to measure dependency at national level. This will reduce the current discretion of researchers when defining FD, as well as the problems derived from different methodological choices needed to measure FD.^66^

 The existence of multiple estimations for the number of dependents in the country hinders the identification of the problem and, consequently, the proposal of solutions. To obtain more accurate estimates of LTC needs at the country level, Mexico needs to move forward from measurements based exclusively on the ENASEM. As noted above, the survey is an important source of information for calculating dependency, but, from a public policy perspective, it has the limitation of being restricted exclusively to older people. On the other hand, its longitudinal nature makes it an important source of information for the study of the dynamics and changes of dependency, but not for the estimation of dependency prevalence, except for the 2001 baseline, and waves with sample refreshments (2012 and 2018).^47^

 In the coming years, Mexico, like other countries, will be facing the challenge of measuring the demand for care services and responding accordingly. We emphasize the need of using consensus-based definitions to collect future data that allows the estimation of FD in older adults as well as in other populations (eg, children and people with dementia) and the periodic updating of the situation of dependents in the country, as a way to advance in the use of evidence-based policy-making.

## Acknowledgements

 The authots thanks to Mariana López-Ortega as well as three anonymous reviewers and the editor for their valuable comments. These suggestions helped improving the original version of the article.

## Ethical issues

 No ethical review was required for the research. The analyses were carried out using publicly available aggregated data.

## Competing interests

 Authors declare that they have no competing interests.

## Authors’ contributions

 PVD and EGB devised the project and conceived the idea. PVD performed the search and generated the list of articles and performed the data analysis. All authors read and approved the final manuscript.

## Supplementary files

Supplementary file 1. Summary of Methodological Decisions Taken to Calculate Dependency Using ENASEM.

## References

[1] Naciones Unidas. División de Población. World Population Prospects - Population Division - United Nations. https://esa.un.org/unpd/wpp/. Accessed May 4, 2018. Published 2019.

[2] Manton KG, Stallard E. Medical demography: interaction of disability dynamics and mortality. In: Martin LG, Preston SH, eds. Demography of Aging. Washington, DC: National Academies Press; 1994. 25144016

[3] Freedman VA, Martin LG, Cornman J, Agree EM, Schoeni RF. Trends in assistance with daily activities: racial/ethnic and socioeconomic disparities persist in the US older population. In: Cutler DM, Wise DA, eds. Health at Older Ages: The Causes and Consequences of Declining Disability Among the Elderly. University of Chicago Press; 2008:411-438. https://www.nber.org/chapters/c11121. Accessed August 19, 2020.

[4] Colombo F, Llena-Nozal A, Mercier J, Tjadens F. Help Wanted?: Providing and Paying for Long-Term Care. Paris: OECD; 2011. 10.1787/9789264097759-en.

[5] Carrera F, Pavolini E, Ranci C, Sabbatini A (2013). Long-term care systems in comparative perspective: care needs, informal and formal coverage, and social impacts in European countries.

[6] Huang C, Soldo BJ, Elo IT (2011). Do early-life conditions predict functional health status in adulthood? the case of Mexico. Soc Sci Med.

[7] World Health Organization (WHO). World Report on Ageing and Health. Geneva: WHO; 2015.

[8] Aranco N, Stampini M, Ibarrarán P, Medellín N. Panorama de Envejecimiento y Dependencia En América Latina y El Caribe. Washington, DC: Inter-American Development Bank; 2018. 10.18235/0000984.

[9] Scheil-Adlung X. Long-Term Care Protection for Older Persons: A Review of Coverage Deficits in 46 Countries. Geneva: ILO, 2015.

[10] Gutierrez-Robledo LM. Relación entre el deterioro funcional, el grado de dependencia y necesidades asistenciales de la población envejecida en México. In: Centro Regional de Investigaciones MultidisciplinariasRelación entre el deterioro funcional el grado de dependencia y necesidades asistenciales de la población envejecida en M, ed. La Población de México Al Final Del Siglo XX, Vol. I; 1998. https://www.crim.unam.mx/web/node/1437. Accessed August 19, 2020.

[11] Hornillos Calvo M, Esteban Dombrid MJ, Urbina Torija J (1998). Influencia de la patología crónica sobre la incapacidad funcional en una población anciana del medio rural. Rev Esp Geriatr Gerontol.

[12] Menéndez J, Guevara A, Arcia N, León Díaz EM, Marín C, Alfonso JC (2005). [Chronic diseases and functional limitation in older adults: a comparative study in seven cities of Latin America and the Caribbean]. Rev Panam Salud Publica.

[13] Soria Romero Z, Montoya Arce BJ (2017). [Aging and factors associated with quality of life for elderly people in State of Mexico]. Papeles Poblac.

[14] Sánchez-García S, Sánchez-Arenas R, García-Peña C (2014). Frailty among community-dwelling elderly Mexican people: prevalence and association with sociodemographic characteristics, health state and the use of health services. Geriatr Gerontol Int.

[15] Peek MK, Ottenbacher KJ, Markides KS, Ostir GV (2003). Examining the disablement process among older Mexican American adults. Soc Sci Med.

[16] Al Snih S, Graham JE, Ray LA, Samper-Ternent R, Markides KS, Ottenbacher KJ (2009). Frailty and incidence of activities of daily living disability among older Mexican Americans. J Rehabil Med.

[17] Villalobos Dintrans P, Bossert TJ, Sherry J, Kruk ME (2019). A synthesis of implementation science frameworks and application to global health gaps. Glob Health Res Policy.

[18] Aguilar-Navarro SG, Amieva H, Gutiérrez-Robledo LM, Avila-Funes JA (2015). Frailty among Mexican community-dwelling elderly: a story told 11 years later The Mexican Health and Aging Study. Salud Publica Mex.

[19] Downer B, Chen NW, Wong R, Markides KS (2016). Self-reported health and functional characteristics of Mexican and Mexican American adults aged 80 and over. J Aging Health.

[20] García Benítez JC. Análisis del Bienestar de Los Adultos Mayores en México. http://conocimientoabierto.flacso.edu.mx/tesis/255. Accessed August 20, 2020. Published 2008.

[21] García-González JJ, García-Peña C, Franco-Marina F, Gutiérrez-Robledo LM (2009). A frailty index to predict the mortality risk in a population of senior Mexican adults. BMC Geriatr.

[22] González CA, Ham-Chande R (2007). [Functionality and health: a typology of aging in Mexico]. Salud Publica Mex.

[23] Grimard F, Laszlo S, Lim W (2010). Health, aging and childhood socio-economic conditions in Mexico. J Health Econ.

[24] Gutiérrez Robledo LM, del Carmen García-Peña M, Jiménez Bolón J. Envejecimiento y Dependencia: Realidades y Previsión Para Los Próximos Años: Documento de Postura. México, DF: Consejo Nacional de Ciencia y Tecnología (CONACYT); 2014.

[25] Instituto Nacional de las Mujeres. Situación de Las Personas Adultas Mayores En México. http://cedoc.inmujeres.gob.mx/documentos_download/101243_1.pdf. Accessed August 20, 2020. Published 2015.

[26] López-Ortega M. Limitación funcional y discapacidad: conceptos, medición y diagnóstico. Una introducción a la situación en México. In: Gutiérrez Robledo L, Stalnikowitz D, eds. Envejecimiento y Salud: Una Propuesta Para un Plan de Acción. SALUD, Secretaría de Salud INGER, Instituto Nacional de Geriatría; 2016. https://bpo.sep.gob.mx/#/recurso/3458. Accessed August 20, 2020.

[27] Lozano Keymolen D, Montoya Arce BJ, Gaxiola Robles Linares SC, Román Sánchez YG (2018). [Functional dependence and its relationship to general mortality in older adults Mexico: 2001-2015]. Poblac Salud Mesoam.

[28] Angel JL, Vega W, López-Ortega M (2017). Aging in Mexico: population trends and emerging issues. Gerontologist.

[29] Manrique-Espinoza B, Salinas-Rodríguez A, Moreno-Tamayo K, Téllez-Rojo MM (2011). [Functional dependency and falls in elderly living in poverty in Mexico]. Salud Publica Mex.

[30] Mejia-Arango S, Gutierrez LM (2011). Prevalence and incidence rates of dementia and cognitive impairment no dementia in the Mexican population: data from the Mexican Health and Aging Study. J Aging Health.

[31] Mejía-Arango S, Miguel-Jaimes A, Villa A, Ruiz-Arregui L, Gutiérrez-Robledo LM (2007). [Cognitive impairment and associated factors in older adults in Mexico]. Salud Publica Mex.

[32] de Oca VM, Hebrero M (2008). Family dynamics, aging, and functional impairment in Mexico. Rev Kairos.

[33] Payne CF (2018). Aging in the Americas: disability-free life expectancy among adults aged 65 and older in the United States, Costa Rica, Mexico, and Puerto Rico. J Gerontol B Psychol Sci Soc Sci.

[34] Smith KV, Goldman N (2007). Socioeconomic differences in health among older adults in Mexico. Soc Sci Med.

[35] Trujillo AJ, Mroz TA, Piras C, Angeles G, Tran N (2012). Caregiving and elderly health in Mexico. Int J Health Serv.

[36] Wong R, Espinoza M, Palloni A (2007). [Mexican older adults with a wide socioeconomic perspective: health and aging]. Salud Publica Mex.

[37] Wong R, Michaels-Obregón A, Palloni A (2015). Progression of aging in Mexico: the Mexican Health and Aging Study (MHAS) 2012. Salud Publica Mex.

[38] Wong R, Michaels-Obregon A, Palloni A (2017). Cohort profile: the Mexican Health and Aging Study (MHAS). Int J Epidemiol.

[39] Avila-Funes JA, Melano-Carranza E, Payette H, Amieva H (2007). [Depressive symptoms as a risk factor for dependence in elderly people]. Salud Publica Mex.

[40] Yahirun JJ, Sheehan CM, Hayward MD (2016). Adult children’s education and parents’ functional limitations in Mexico. Res Aging.

[41] Barrantes-Monge M, García-Mayo EJ, Gutiérrez-Robledo LM, Miguel-Jaimes A (2007). [Functional dependence and chronic disease in older Mexicans]. Salud Publica Mex.

[42] Beltrán-Sánchez H, Pebley A, Goldman N (2017). Links between primary occupation and functional limitations among older adults in Mexico. SSM Popul Health.

[43] Díaz de León González E, Gutiérrez Hermosillo H, Martinez Beltran JA (2016). Validation of the FRAIL scale in Mexican elderly: results from the Mexican Health and Aging Study. Aging Clin Exp Res.

[44] Díaz de León González E, Tamez Pérez HE, Gutiérrez Hermosillo H, Cedillo Rodríguez JA, Torres G (2012). [Frailty and its association with mortality, hospitalization and functional dependence in Mexicans aged 60-years or older]. Med Clin (Barc).

[45] Díaz-Venegas C, De La Vega S, Wong R (2015). Transitions in activities of daily living in Mexico, 2001-2012. Salud Publica Mex.

[46] Dorantes-Mendoza G, Avila-Funes JA, Mejía-Arango S, Gutiérrez-Robledo LM (2007). [Factors associated with functional dependence in older adults: a secondary analysis of the National Study on Health and Aging, Mexico, 2001]. Rev Panam Salud Publica.

[47] University of Texas Medical Branch. MHAS Publications. http://www.mhasweb.org/Publications.aspx. Accessed August 20, 2020. Published 2012.

[48] VanSant AF (2006). The international classification of functioning, disability and health. Pediatr Phys Ther.

[49] Instituto Nacional de Estadística y Geografía (INEGI). XII Censo General de Población y Vivienda, 2000. Aguascalientes: INEGI; 2000.

[50] Federal Ministry of Health. Report by the Advisory Board to Review the Definition on Need for Long-Term Care. Germany: Federal Ministry of Health; 2009.

[51] Jeon B, Kwon S (2017). Health and long-term care systems for older people in the republic of Korea: policy challenges and lessons. Health Syst Reform.

[52] Jefatura del Estado (España). Ley 39/2006, de 14 de diciembre, de Promoción de la Autonomía Personal y Atención a las personas en situación de dependencia. Boletín Oficial del Estado. BOE-A-2006-21990. https://www.boe.es/buscar/pdf/2006/BOE-A-2006-21990-consolidado.pdf. Accessed August 19, 2020.

[53] Servicio Nacional del Adulto Mayor (SENAMA). Estudio Nacional de La Dependencia En Las Personas Mayores. Santiago: SENAMA; 2010. http://www.microdatos.cl/Documentos/docto_publicaciones/Estudio_Dependencia_Personas_Mayores.pdf. Accessed May 17, 2018.

[54] Villalobos Dintrans P (2017). [Aging and long-term care in Chile: challenges in the OECD context]. Rev Panam Salud Publica.

[55] Sistema de Cuidados (Uruguay). Baremo de dependencia formulario para Sistema de Cuidados. https://www.gub.uy/sistema-cuidados/sites/sistema-cuidados/files/documentos/publicaciones/formulario-de-aplicacion-baremo-de-dependencia.pdf.

[56] Orimo H (2006). [Reviewing the definition of elderly]. Nihon Ronen Igakkai Zasshi.

[57] Sanderson WC, Scherbov S (2010). Demography Remeasuring aging. Science.

[58] Dintrans PV (2018). Is aging a problem?: Dependency, long-term care, and public policies in Chile. Rev Panam Salud Publica.

[59] Villalobos Dintrans P (2019). [Dependency in Chile Advances and challenges]. Rev Med Chil.

[60] Sistema de Cuidados Uruguay. Cuidades rinde cuentas; 2017. https://www.gub.uy/sistema-cuidados/sites/sistema-cuidados/files/2020-03/Cuidados%20rinde%20cuentas_Agosto%202017.pdf. Accessed August 20, 2020.

[61] Instituto Nacional de Salud Pública. Encuesta Nacional de Salud y Nutrición 2018. https://ensanut.insp.mx/encuestas/ensanut2018/descargas.php. Accessed August 19, 2020.

[62] Villalobos Dintrans P (2020). Designing long-term care systems: elements to consider. J Aging Soc Policy.

[63] Tabak RG, Khoong EC, Chambers DA, Brownson RC (2012). Bridging research and practice: models for dissemination and implementation research. Am J Prev Med.

[64] Instituto Nacional de Estadística y Geografía (INEGI). Encuesta Nacional de Ingresos y Gastos de los Hogares. Aguascalientes: INEGI; 2016.

[65] Kingdon JW. Agendas, Alternatives, and Public Policies. Boston: Longman; 2011.

[66] Villalobos Dintrans P, Chaumont C (2017). Examining the relationship between human resources and mortality: the effects of methodological choices. Int J Public Health.

