# Supplementary material for: Functional Dependency in Mexico: Measurement Issues and Policy Challenges
Source: Int J Health Policy Manag. 2020 Dec 19;11(7):1017–23. doi: 10.34172/ijhpm.2020.248 (PMC9808163; doi:10.34172/ijhpm.2020.248)
Supplement: Supplementary file 1 — Summary of Methodological Decisions Taken to Calculate Dependency Using ENASEM. [file ijhpm-11-1017-s001.pdf]

Supplementary file 1. Summary of Methodological Decisions taken to calculate dependency using the ENASEM

Reference	Activities Used	Criteria to Define FD	Reported Result
[13] Aguilar-Navarro et al. (2015)	BADL (5): Katz index (bathing, walking, moving from bed to chair, continence, eating)<br>IALD (4): Lawton and Brody scale (taking medicines, managing money, shopping, grooming)	Declares difficulty in at least one activity	Prevalence<br>BADL: 3%<br>IADL: 10.1%
[3] Aranco et al. (2018)	BADL (5): bathing, toileting, eat/dink, dressing, changing positions/ getting in and out of the bed	Declares difficulty in at least one activity	Prevalence for type of activity<br>People with FD: 2,417,000
[19] Díaz de León González et al. (2012)	BADL (6): bathing, dressing, toileting, eating, continence<br>IADL (4): preparing meals, shopping, taking medicines, managing money	Sum of BADL and IADL that require help	Report incidence instead of prevalence
[20] Díaz-Venegas C et al. (2015)	BADL (5): Katz index (bathing, dressing, eating, toileting, moving in and out of bed)	Declare need for help of not being able to perform at least one activity	Number of BADL limitations:<br>0: 72.8<br>1-2: 20.4<br>3+: 6.8
[21] Dorantes-Mendoza et al. (2007)	BADL (5) Katz index (bathing, dressing, toileting, moving inside the house, eating)<br>Continence was analyzed as a binary variable, independently from the other BADL.<br>IADL (4): Lawton and Brody (preparing meals, taking medicines, shopping, managing money)	Declares difficulty and need for help in for performing at least one of these activities.	Prevalence<br>BADL dependents: 7.3%<br>IADL dependents: 8.4%
[34] Mejía-Arango and Gutierrez (2011)	BADL (5): walking, bathing, eating, going to bed, toileting<br>IADL (4): preparing a hot meal, shopping, taking medicines, managing money	People that requires help in at least one or more BADL and/or two or more IADL	Reports prevalence jointly with cognitive impairment and FD (3.3%)
[36] Montes de Oca and Hebrero (2008)	BADL (5): walking, bathing, eating, going to bed, toileting<br>IADL (4): preparing a hot meal, shopping, taking medicines, managing money	Declares difficulty in at least one activity	Prevalence<br>BADL limitation: 15%<br>IADL limitation: 13%<br>BADL limitation only: 7.7%<br>IADL limitation only: 6.0%<br>BADL + IADL limitation: 7.7%
[28] Trujillo et al. (2012)	BADL (6): bathing, dressing, toileting, walking, going in and out from chair, eating<br>IADL (6): preparing meals, shopping, managing money, using the phone, grooming (light and heavy duties)	BADL index: scale from 0 to 5<br>IADL index: scale from 0 to 15	Number of people with FD<br>BADL: Men Women<br>60-70: 4,809 4,842<br>70-85: 4,659 4,571<br>85+: 3,864 3,311<br>IADL: Men Women<br>60-70: 12,037 11,001<br>70-85: 10,902 10,196<br>85+: 9,133 9,992
